# Ultrasound-guided hydrodilatation for adhesive capsulitis of the hip is a safe and effective treatment

**DOI:** 10.1007/s00264-020-04909-y

**Published:** 2021-01-18

**Authors:** Byung-Ho Yoon, Jae-Chan Shim, MinKi Lee, Hyoung-Keun Oh, Yerl-Bo Sung, Suk Kyu Choo

**Affiliations:** 1 Department of Orthopaedic Surgery, Inje University College of Medicine, Seoul Paik Hospital, Seoul, Korea; 2 Department of Radiology, Inje University College of Medicine, Sanggye Paik Hospital, Seoul, Korea; 3 Department of Orthopedic Surgery, Inje University College of Medicine, Ilsan Paik Hospital, Goyang-si, Gyeonggi-do Korea; 4 Department of Orthopaedic Surgery, Inje University College of Medicine, Sanggye Paik Hospital, Seoul, Korea

**Keywords:** **Hip**, **Adhesive capsulitis**, **Capsule**, **Ultrasound**, **Hydrodilatation**

## Abstract

**Purpose:**

Adhesive capsulitis of the hip (ACH) is likely that this condition had been previously encountered, but easily unrecognised. We investigated the clinical features of patients with ACH, the efficacy of ultrasound-guided intra-articular hydrodilatation, and the patients’ prognosis.

**Methods:**

We enrolled 84 patients (93 hips) who visited the outpatient clinic from August 2018 to November 2019. ACH was diagnosed by restricted range of motion and sharp pain when turning with the affected leg fixed on the ground. We evaluated patient demographics and associated intra-articular pathologies found on magnetic resonance angiography (MRA) images. Injections were performed twice at two week intervals with a mixture of 0.5% lidocaine (25 mL) and triamcinolone (40 mg; 1 mL) with capsular distension under ultrasound guidance. Patients were assessed before and after treatment using a visual analogue scale (VAS), hip disability and osteoarthritis outcome score (HOOS), hip range of motion (ROM), and distance from floor to knee (DFK) when sitting in the cross-legged position.

**Results:**

On MRA, 18 patients had abnormal findings (eight labral tears, seven abductor tendinosis, three primary arthrosis). The mean VAS decreased from 7.1 ± 1.1 to 0.8 ± 0.9 after the last injection, and the HOOS improved in all subsets. The mean DFK decreased from 17.9 ± 4.8 to 9.7 ± 2.8 cm, and passive ROM showed improvement, especially in flexion and rotation. In seven patients, symptom recurrence was reported a mean of 4.1 months after the latest injection, but no independent risk factor for recurrence was identified.

**Conclusion:**

Based on these current observations, patients with ACH may receive relief from hip joint pain and experience improved function with a timely diagnosis and effective treatment.

## Introduction

Adhesive capsulitis (AC) is a well-known clinical disease that has been studied in the shoulder but can occur in any joint [1, 2]. Caroit et al. [3] first introduced the concept of adhesive capsulitis of the hip (ACH), which is particularly characterised by a painful decrease in active and passive range of motion, especially rotation [4, 5]. Byrd and Jones also describe ACH as a clearly identifiable entity, and they describe it as being more common than would be suggested by the paucity of literature on this topic [6]. The diagnostic criteria of ACH are still not well-defined, and its diagnosis tends to be subjective [7–9]. Furthermore, previous studies on ACH included many cases secondary to underlying conditions such as degenerative arthritis or synovial chondromatosis in addition to primary cases, which makes it difficult to extrapolate a treatment strategy based on these articles [6, 10, 11].

Currently, many treatment options are utilised for AC, including oral nonsteroidal anti-inflammatory drugs, physical therapy, corticosteroid injection, pressure dilation, and arthroscopic surgery [12]. The capsular distension method has also been employed, in which a large volume of fluid is injected into the joint to separate the adherent synovium and stiffened capsule by hydropressure. Several previous studies showed excellent efficacy of this procedure, which is now widely used in the treatment of AC, especially shoulder AC [12–14]. It is easy to perform, requires no radiation, and is less invasive than surgery. However, the effectiveness of hydrodilatation on ACH has rarely been discussed in the literature [13, 15]. To our knowledge, there are few case reports on the treatment efficacy of ultrasound (US)-guided hydrodilatation, and a detailed prospective study of this treatment has not been performed [10, 16].

Herein, we report the results of a prospective evaluation of US-guided hydrodilatation for ACH. The purposes of this study were (1) to describe the clinical characteristics of patients who manifest ACH and (2) to determine the outcomes of this procedure using a prospective study. We hypothesised that US-guided injection with capsular distension would improve passive hip range of motion (ROM), general clinical outcome measures, and pain scales.

## Materials and methods

### Diagnosis and patient selection

We prospectively enrolled patients from August 2018 to November 2019, and ACH was diagnosed based on the patients’ medical history and physical examination and radiologic findings. Institutional ethical committee approval was obtained prior to beginning the study, and informed consent was obtained from all study participants.

Patients over age 18 were included when they met the following two criteria: (1) the sum of the three passive ROM measurements was reduced by > 30% compared to that on the contralateral side—forward flexion (FF), internal rotation (IR), and external rotation (ER) at 90° knee flexion in the supine position. If both hips were involved, the sum of the ROM measurements was less than 160°, which is 70% of the normal range [4, 5, 7]. Passive ROM was performed to maximum patient toleration or mechanical block. (2) Sharp hip pain when turning while weight-bearing with the affected leg [4, 17]. All patients were given a preliminary course of analgesics for three weeks for pain relief. Only patients who did not respond to analgesics and continued to have severe pain were administered the intra-articular injection.

We excluded patients with the following diseases based on X-ray or MRI: (1) significant hip joint arthritis (plain radiographic finding of Kellgren-Lawrence grade > 2) [18], (2) femoroacetabular impingement (large cam and pincer lesion causing joint restriction), (3) osteonecrosis of the femoral head, and (4) synovial chondromatosis. We also excluded patients with a history of systematic rheumatic disease, a chronic pain condition (such as fibromyalgia), side effects to local anaesthetics, presence or suspicion of infection, or major trauma or surgery of the hip joint. In addition, patients who were taking warfarin or nonvitamin K-dependent antagonist oral anticoagulants were excluded owing to the risk of haematoma formation after injection.

### Identification of patient demographics and concomitant pathology

First, we assessed patient information including sex, age, diabetes, thyroid disease, and previous history of treatment for other diseases because idiopathic AC commonly occurs in patients with comorbidities such as hormonal, cardiac, or neurologic disorders. Data on baseline symptoms were also collected. All patients underwent magnetic resonance arthrography (MRA) to rule out other causes of pain that were described in the exclusion criteria and to identify concomitant lesions. Concomitant pathologies included primary arthrosis (Kellgren-Lawrence grade 1), acetabular labrum lesions, ligamentum teres lesions, and abductor tendinosis.

### Ultrasound-guided intra-articular injection techniques

**Fig. 1:**
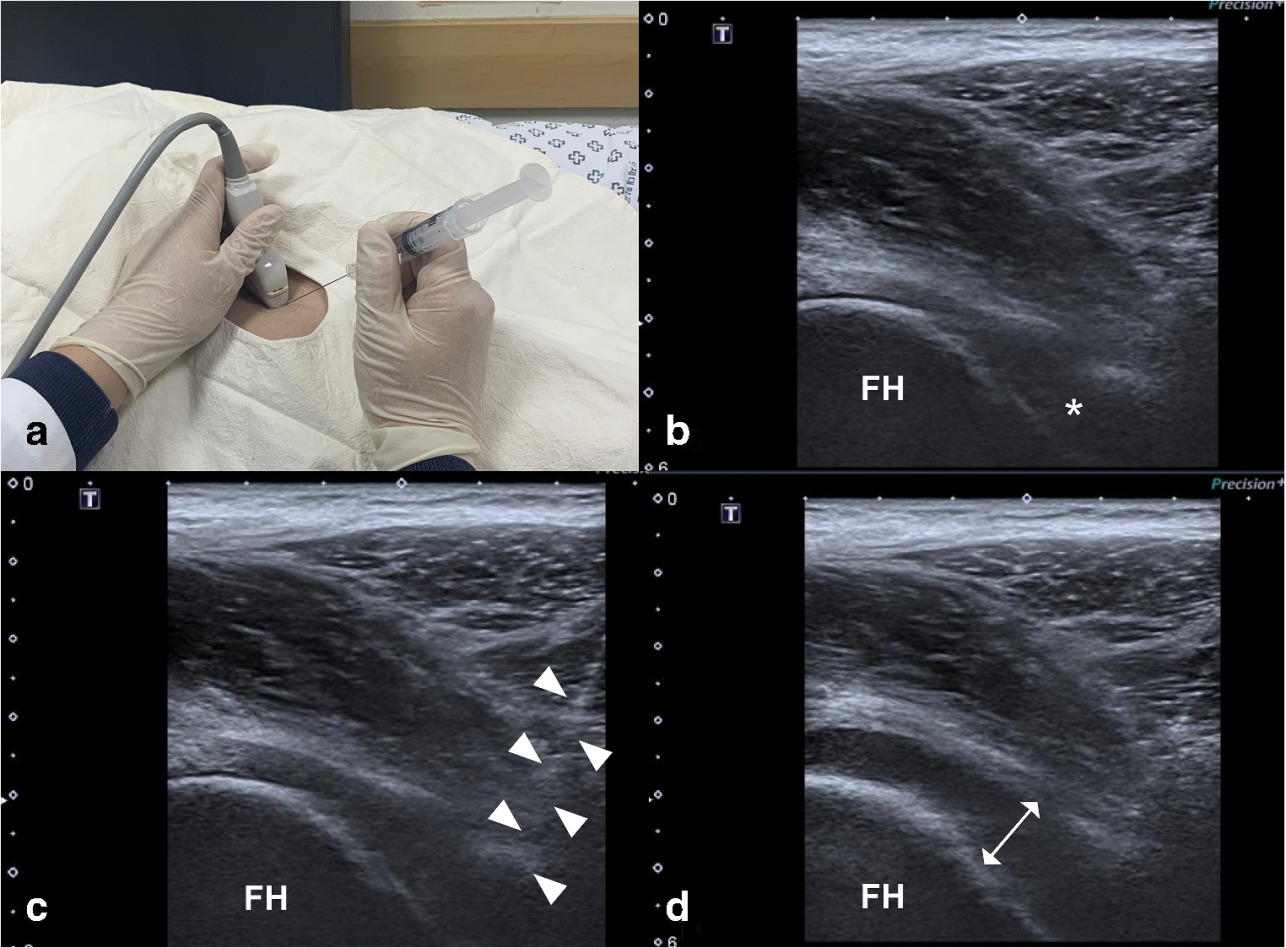
Images of the sagittal-oblique plane parallel to the femoral neck in a 47-year-old male patient in the supine position. Note the path of the needle to reach the joint cavity (white arrowheads). (A) Ultrasound-guided intra-articular injection was performed by placing the curvilinear transducer firmly over the area of the femoral head-neck junction in the long axis and slightly oblique view (anterolateral approach). (B) The anterior recess (*) of the hip joint over the femoral neck (normally about 4 to 6 mm thick) is targeted. (C) The needle is visualised as a hyperechoic line (arrow) and is targeted to the anterior synovial recess located at the neck of the femur. Two millilitres of fluid was injected to confirm the location of the needle tip. (D) After ultrasound-guided injection, maximum tolerable distension was achieved without rupturing the capsule (double arrow). FH, femoral head; AR, anterior recess of hip joint

All US-guided intra-articular (IA) injections were performed by one orthopaedic surgeon with over ten years of relevant experience using a linear 7-MHz probe in grey scale and Doppler mode. Patients were placed in a supine position on a table with the heels together and the legs externally rotated 10–20° degrees. An antero-inferior longitudinal approach was used. A spinal needle (23 G, 9.5 cm) was inserted up to the bone through the rectus and iliopsoas muscle and capsule with direct tracing of the progression of the needle (Fig. 1A, B). After bony cortex contact, the needle was retracted 3–4 mm in order to avoid engaging the tip in the posterior synovial layer and to facilitate the tip placement inside the anterior joint recess (Fig. 1C). Next, 0.5% lidocaine (25 mL) with triamcinolone (40 mg; 1 mL) was administered, and capsular distention was confirmed (Fig. 1D) [15, 19].

All patients received two US-guided IA injections at an interval of two weeks (a total of two injections). Additionally, acetaminophen or ice massage was permitted if the patients complained of pain at the injection site. No specific simple exercise program or physical therapy was performed during the injection interval.

### Assessments of patient outcomes

The same outcome assessor evaluated all patients at baseline and two weeks after the second injection. The outcomes were pain score that was assessed by a visual analogue scale (VAS) (0, no pain; 10, extreme pain), passive ROM of the hip, distance from floor to knee (DFK) when sitting in the cross-legged position, and the hip disability and osteoarthritis outcome score (HOOS). Each subscale of the HOOS is scored on a scale of 1 to 100, with a score of 100 indicating no problems (best outcome) [20]. The secondary outcome was the presence of complications.

One physician assisted the patient to achieve maximum passive motion, and the other measured passive ROM in all directions using a Dualer IQ inclinometer. Measurements were repeated two times, and the results were expressed as averages. Finally, any adverse events including fat atrophy, infection, facial rash, and local secondary crystalline synovitis neuropraxia were monitored, recorded, and reported by the investigators at each visit.

The patients were followed up for six months after treatment. The patients were followed up by clinical visits or telephone interviews.

### Statistical analysis

We calculated the sample size based on a reduction of 20% in the HOOS pain score. A similar trial assumed a mean HOOS pain score at presentation of 60, indicating that 12 is a clinically significant decrease in the score. Using an alpha of 5% and a power of 80%, the required sample size was 50 patients, allowing for a 10% attrition/noncompliance rate.

Because all variables showed normal distribution, a paired *t* test was used to compare all outcome variables between pre-treatment and latest follow-up. Then, we divided our cohort into two groups according to symptom recurrence after treatment. To determine associated risk factors for symptom recurrence, a binary logistic regression analysis was assessed between patient characteristics including age, sex, body mass index, symptom duration, bilaterality, diabetes mellitus, thyroid disease, cardiovascular disease, and smoking. All analyses were performed using STATA (version 14.0; Stata Corporation, College Station, TX, USA).

## Results

### General characteristics of included patients

Ninety-six patients were originally considered, and 84 patients were enrolled after excluding for the following reasons: severe joint or posttraumatic arthritis (*n* = 5), previous hip surgery (*n* = 2), warfarin use (*n* = 1), or refusal to participate (*n* = 4). A total of 84 patients with a mean age of 55.5 years were included, and both hips were affected in nine (10.7%) patients. The mean symptom duration (the mean time interval from the development of symptoms and the first visit) was 5.3 months (range 3–13) and of the MRA studies, eight (9.5%) showed evidence of labral pathology and seven (8.3%) showed abductor tendinosis. Baseline characteristics are summarised in Table 1. Fifteen patients had a history of diabetes with a mean of 7.2 years of treatment, and one patient had been treated for angina.

**Table 1:** Baseline characteristics of patients with adhesive capsulitis of hip

Number of patients (hips)	84 patients (93 hips)
Age (year,), mean (SD)	55.5 (8.9)
Sex (men/women)	46/38
Bilaterality	9 (10.7%)
BMI (kg/m^2^), mean (SD)	22.7 (3.5)
Symptom duration (month), mean (SD)	5.3 (5.6)
Night sleep disturbance	13 (15.4%)
Diabetes	15 (17.8%)
Thyroid disease (hypothyroidism and partial thyroidectomy)	3 (3.6%)
Angina	1 (1.2%)
Smoking	24 (28.5%)
Concomitant pathology	18 (21.4%)
Primary arthrosis (Kellgren-Lawrence grade 1)	3 (3.6%)
Labral tear, suggested	8 (9.5%)
Gluteus minimus or medius tendinosis	7 (8.3%)
Symptom recurrence within 6 months	7 (8.3%)

The data is presented as mean (standard deviation) or number (%).

### Change in pain severity by visual numeric scale and disability index

The average VAS score decreased from 7.1 (range, 5–9) before the procedure to 0.8 (range, 0–3; *p* < 0.001) at the latest follow-up. The patient-reported outcome as assessed by the HOOS also improved in all subsets (*p* < 0.001; Table 2). The mean true DFK decreased from 17.9 ± 4.8 cm (range, 13–30 cm) to 9.7 ± 2.8 cm (range, 5–15 cm; *p* < 0.001). Passive range of motion before and after the procedure showed improvement especially in FF, ER, and IR (*p* < 0.001; Table 2).

**Table 2:** Comparison of mean pre- and post-operative parameters of patients

Variable	Pre-injection	Last follow-up	*P* value
VAS	7.1 ± 1.1	0.8 ± 0.9	< 0.001
DFK	17.9 ± 4.8	9.7 ± 2.8	< 0.001
Range of motion (degree)			< 0.001
Flexion	97.1°± 5.4°	117.2°± 7.5°	< 0.001
Extension	15.2°± 12.5	25.3°± 2.9°	< 0.001
Internal rotation	11.1°± 1.8	26.4°± 4.3°	< 0.001
External rotation	20.7°± 1.6°	42.9°± 4.6°	< 0.001
Abduction	32.8°± 0.9°	40.3°± 0.2	< 0.001
Adduction	19.3°± 0.5°	20.3°± 0.3°	0.110
HOOS			
Symptom	77.5 ± 8.6	88.8 ± 5.7	< 0.001
Pain	78.3 ± 6.4	93.5 ± 5.3	< 0.001
ADL	85.3 ± 6.1	96.2 ± 4.7	< 0.001
Sports	80.1 ± 10.2	93.4 ± 9.7	< 0.001
QoL	65.3 ± 13.4	86.8 ± 5.2	< 0.001

*Data are provided as mean ± SD

ADL, activities of daily living; DFK, distance from floor to knee on sitting in cross-legged position; HOOS, hip disability and osteoarthritis outcome score; QoL, quality of life; VAS, visual analogue scale

### Adverse events and symptom recurrence

No patients reported severe adverse events related to the injection, but two patients revisited the clinic with moderate pain after the first injection on the same day. Intramuscular painkiller injection successfully relieved the pain. In seven (8.3%) patients, recurrence of symptoms was reported after a mean of 4.1 months after the latest injection. They underwent the injection protocol again, and the symptoms were relieved. No significant risk factor for symptom recurrence was found (Table 3).

**Table 3:** The association between symptom recurrence and other variables

	Logistic regression analysis		
Variables	Coefficient	95% CI	*p*
Age (year)	0.937	0.699; 1.257	0.665
Sex (men/women)	0.977	0.775; 1.232	0.849
Bilaterality	1.727	0.512; 3.213	0.175
Body mass index (kg/m^2^)	1.080	0.622; 1.875	0.784
Symptom duration (month)	0.678	0.174; 2.330	0.531
Night sleep disturbance	1.537	0.775; 2.232	0.494
Diabetes	3.162	0.081; 12.46	0.537
Thyroid disease (hypothyroidism and partial thyroidectomy)	NA		
Angina	NA		
Smoking	1.326	0.004; 41.34	0.916
Concomitant pathology	NA		
Primary arthrosis (Kellgren-Lawrence grade 1)			
Labral tear, suggested			
Gluteus minimus or medius tendinosis			

CI, confidence interval; NA, non-available

## Discussion

ACH is a condition of unknown aetiology characterised by decreased active and passive range of motion, which is painful [4, 6, 7]. Its clinical symptoms are very similar to those of frozen shoulder; therefore, ACH has been referred to as frozen hip [3]. Despite the similarities between these diseases, treatment options for ACH are not well established, and evidence for their effectiveness is very limited. This study is the first large case series of ACH treated with hydrodilatation that assessed the rate of recurrence.

ACH is commonly diagnosed as a combination of pain that causes joint stiffness, mainly affecting rotation and flexion, and pain that is exacerbated by weight-bearing or activity [1, 4, 5, 11]. To accurately diagnose ACH, ROM must be evaluated; the pelvis must be fixed while measuring the hip ROM so that pelvic flexion or rotation does not add to the hip ROM. Some clinicians have used a significant reduction in fluid capacity as a diagnostic criterion for ACH, but a patient may have ACH without a significant reduction in volume [6–8]. We first included our patients based on the clinical presentation, and then radiologic findings including X-ray and MRA were used to rule out all other possible conditions, structural pathology, or concomitant pathology.

We observed clear improvement in patient-reported outcomes but noticed that their pre-treatment baseline scores were not as low as those of femoroacetabular impingement, dysplastic hip, or severe arthritic hip [21, 22]. The patients usually present with pain, especially with extreme external rotation or abduction, so they usually complain of difficulty in crossing the leg or sitting in certain positions such as getting in and out of a car. ACH only slightly restricts movement, and gait difficulty may or may not be present [1, 6, 10]. Adhesive capsulitis is underdiagnosed because the hip joint can sustain range of motion loss without significant disability, whereas even a mild loss of motion in the shoulders can result in significant difficulty performing routine activities of daily living [3, 6, 8, 23].

According to earlier reports, spontaneous resolution of symptoms is possible but unpredictable, requiring over 18 months in most cases [7, 10, 16, 17]. IA injections with capsular distension are easily performed, and many reviews support hydrodilatation as a treatment modality to improve short-term pain and function [16, 19, 24]. Thus, it is a good nonsurgical option for patients with ongoing pain and disability and for whom complete spontaneous resolution cannot be guaranteed. Previous studies on ACH provide little evidence to determine whether capsule rupture must be achieved in order for the procedure to be successful [25, 26]. Obviously, it is impossible to rupture the hip joint capsule, which is much stronger and denser than the shoulder capsule; thus, we focused on capsular distension, which is the most important [2]. US guidance improved the accuracy of the injections and avoided the side effects associated with extra-articular leakage and injury of adjacent structures. No adverse effects were observed in any patient. Two male patients reported moderate-to-severe pain after the first injection, so they revisited the outpatient clinic on the same day. These patients had very restricted motion, and the clinician felt much pressure during the injection. The pain easily subsided after the intramuscular injection, but patients should be counselled regarding the possibility of short-term increased pain after injection when presenting with a severe contracture.

In our cohort, seven (8.3%) patients returned with symptom recurrence. We are not aware of any reports on the treatment of recurrent ACH, so we repeated the US-guided IA injection, and it benefitted all patients. No association between symptom recurrence and comorbidities typically associated with AC, such as diabetes, was demonstrated in our cohort [27, 28]. Abnormal findings in the labrum, ligamentum teres, or capsular ligament were detected on MR images, but they were not interpreted to be related to the symptom recurrence. We observed that 13 patients (15.4%) suffered from night sleep disturbance (NSD) pain that involved difficulty lying on the affected side, and some patients awoke from sleep when their affected limb was toward the other side. Correspondingly, symptom severity and duration, including NSD, had no association with recurrence (Table 3).

Given that we observed favourable outcomes as an immediate clinical response, arthroscopic surgery should be considered for underlying identifiable causes of capsular construction such as synovial chondromatosis or severe femoroacetabular lesion [5–7]. A more structured and sequential method for treating ACH is warranted to understand when arthroscopy is indicated.

This study has several limitations. First, the stages of adhesive capsulitis were not classified. ACH is characterised by (a) synovial inflammation in the acute stage with a painful decrease in active and passive range of motion and (b) capsular fibrosis in the chronic stages with significant limitation of joint motion. However, no reports have described distinct stages of ACH in the hip joint. We suspect that most of our patients might have had acute ACH because of the obtained symptom relief without aggressive physical therapy or manipulation under anaesthesia. Second, we did not employ any active stretching exercises following injection to augment our treatment. The recurrence rate might have been lowered if continuous motion exercises had been applied. Third, corticosteroids, which reduce inflammation and alleviate pain, are the major mechanism behind our improved clinical outcomes. Thus, studies are needed to compare the effect corticosteroids alone and hydrodilatation with corticosteroids in patients with ACH.

## Conclusion

On the basis of our study, US-guided hip injection with hydrodilatation can be recommended as a safe and effective way of obtaining symptom relief in patients with ACH. However, further research is required to understand the pathophysiology behind the disease to identify the reasons for recurrence so that stage-appropriate ACH treatment can be recommended.

## Data Availability

Not applicable.
